# Sphingoid Base Metabolism in Yeast: Mapping Gene Expression Patterns Into Qualitative Metabolite Time Course Predictions

**DOI:** 10.1002/cfg.106

**Published:** 2001-10

**Authors:** Tomas Radivoyevitch

**Affiliations:** Department of Epidemiology and Biostatistics BRB G-19 Case Western Reserve University Cleveland OH 44106 USA

## Abstract

Can qualitative metabolite time course predictions be inferred from measured mRNA expression patterns? Speaking against this possibility is the large number of ‘decoupling’ control points that lie between these variables, i.e. translation, protein degradation, enzyme inhibition and enzyme activation. Speaking for it is the notion that these control points might be coordinately regulated such that action exerted on the mRNA level is informative of action exerted on the protein and metabolite levels. A simple kinetic model of sphingoid base metabolism in yeast is postulated. When the enzyme activities in this model are modulated proportional to mRNA expression levels measured in heat shocked yeast, the model yields a transient rise and fall in sphingoid bases followed by a permanent rise in ceramide. This finding is in qualitative agreement with experiments and is thus consistent with the aforementioned coordinated control system hypothesis.


**Comparative and Functional Genomics**
*Comp Funct Genom* 2001; **2**: 289–294.
**DOI:** 10.1002/cfg.106

**Short Communication**

### Sphingoid base metabolism in yeast: mapping gene expression patterns into qualitative metabolite time course predictions

Tomas Radivoyevitch*
*Department of Epidemiology and Biostatistics, Case Western Reserve University, Cleveland, OH 44106, USA*

**Correspondence to:
T. Radivoyevitch, Department of Epidemiology and Biostatistics BRB G-19, Case Western Reserve University, Cleveland, OH 44106, USA.
E-mail: radivot@hal.cwru.edu*

Received: 8 July 2001
Accepted: 13 August 2001

### Abstract

**Can qualitative metabolite time course predictions be inferred from measured mRNA expression patterns? Speaking against this possibility is the large number of 'decoupling' control points that lie between these variables, i.e. translation, protein degradation, enzyme inhibition and enzyme activation. Speaking for it is the notion that these control points might be coordinately regulated such that action exerted on the mRNA level is informative of action exerted on the protein and metabolite levels. A simple kinetic model of sphingoid base metabolism in yeast is postulated. When the enzyme activities in this model are modulated proportional to mRNA expression levels measured in heat shocked yeast, the model yields a transient rise and fall in sphingoid bases followed by a permanent rise in ceramide. This finding is in qualitative agreement with experiments and is thus consistent with the aforementioned coordinated control system hypothesis. Copyright © 2001 John Wiley & Sons, Ltd.**

**Keywords:** gene expression; sphingoid base; ceramide; heat shock; yeast

### Introduction

Sphingoid base metabolites are important second messengers in cellular responses to stress (Hannun, 1996). In yeast, sphingoid base metabolism is important not only as a model of its mammalian counterpart (Schneiter, 1999; Dickson, 1998), but also in and of itself as a system that contains several anti-fungal drug targets (Nagiec *et al.*, 1997; Dickson and Lester, 1999).

It is hypothesized here that cells react to stress through a coordinated control effort whereby action on the mRNA level is qualitatively informative of action on the protein and metabolite levels. For example, one can speculate that mRNA time courses lead enzyme activity time courses when metabolism is controlled predominantly on the mRNA level, and, in the interests of producing less enzyme when less enzyme is demanded, that mRNA time courses lag behind enzyme activity time courses when control is mediated predominantly on the level of metabolites. In other words, one can speculate that there is an associative (not necessarily causal) correlation between mRNA time courses and enzyme activity time courses. If so, qualitative metabolite time course predictions may be inferable from measured mRNA time courses. This possibility is explored here for sphingoid base metabolism in heat shocked yeast (Eisen *et al.*, 1998).

### Methods and results

A simplified schematic of yeast sphingoid base metabolism is shown in Figure 1. In this model, fatty acid synthase (Fas = Fas1/2) creates palmitate ($C_{16}$) which reacts in two ways. It condenses with serine (via serine palmitoyl transferase, Spt = Lcb1/2) to form the sphingoid bases dihydrosphingosine

Copyright © 2001 John Wiley & Sons, Ltd.

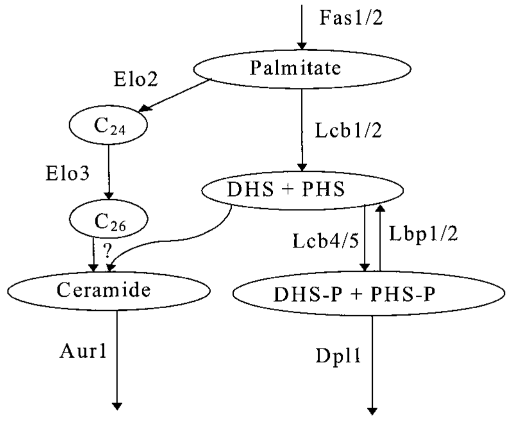

**Figure 1.** Sphingoid base metabolism in yeast. The metabolite pools are palmitate, $C_{24}$, $C_{26}$, DHS and PHS (pooled), DHS-P and PHS-P (also pooled), and ceramide, see text

(DHS) and phytosphingosine (PHS), and it condenses repeatedly with malonyl-CoA (via Elo2 and Elo3) to form the elongation products $C_{24}$ and $C_{26}$. Here $C_{16}$, $C_{24}$ and $C_{26}$ are 16-, 24- and 26-carbon saturated fatty acids, respectively. The sphingoid bases can be phosphorylated by sphingoid base kinase (Sk = Lcb4/5) to form DHS-P and PHS-P. These phosphorylated forms can be degraded by dihydrosphingosine phosphate lyase (Dpl), or dephosphorylated back to DHS and PHS by long chain base phosphatase (Lbp = Lbp1/2)—the terms 'sphingoid base' and 'long chain base' are used here for any one of DHS, PHS, DHS-P and PHS-P. Free (ie. unphosphorylated) sphingoid bases can react with $C_{26}$ to form ceramide via ceramide synthase (Cs), which has not yet been cloned. Ceramide is removed from the system by inositolphosphorylceramide synthase (Aur1). As most of the enzymes in this system have yet to be purified, reaction rate laws are not known and must be assumed. In the equations that follow, substrate concentrations are raised to the power 0.5 as an *a priori* power-law approximation to Michaelis-Menten kinetics (Voit, 2000).

$$\frac{d}{dt}C_{16} = Fas - (.5+\phi/2) * Spt * C_{16}^{0.5} - (.5-\phi/2) * Elo2 * C_{16}^{0.5} \tag{1}$$

$$\frac{d}{dt}C_{24} = (.5-\phi/2) * Elo2 * C_{16}^{0.5} - (.5-\phi/2) * Elo3 * C_{24}^{0.5} \tag{2}$$

$$\frac{d}{dt}C_{26} = (.5-\phi/2) * Elo3 * C_{24}^{0.5} - (.5-\phi/2) * Cs * C_{26}^{0.5} * DHS^{0.5} \tag{3}$$

$$\frac{d}{dt}DHS = (.5+\phi/2) * Spt * C_{16}^{0.5} - (.5-\phi/2) * Cs * C_{26}^{0.5} * DHS^{0.5} - \phi * Sk * DHS^{0.5} + \psi * (Lbp * DHSP^{0.5} - Sk * DHS^{0.5}) \tag{4}$$

$$\frac{d}{dt}DHSP = (\phi+\psi) * Sk * DHS^{0.5} - \phi * Dpl1 * DHSP^{0.5} - \psi * Lbp * DHSP^{0.5} \tag{5}$$

$$\frac{d}{dt}CER = (.5-\phi/2) * Cs * C_{26}^{0.5} * DHS^{0.5} - (.5-\phi/2) * Aur1 * CER^{0.5} \tag{6}$$

In these equations, *DHS* represents the sum of DHS and PHS, *DHSP* represents the sum of DHS-P and PHS-P, and *CER* is ceramide. The initial conditions for these metabolites are $C_{16}(0)=1$, $C_{24}(0)=1$, $C_{26}(0)=1$, $DHS(0)=1$, $DHSP(0)=1$, and $CER(0)=1$. By design, this initial state is also the steady state when the enzyme activities are unity. The enzyme activities and the main flux coming into the system take unit values at $t=0$, i.e. just before heat shock. Thus, the fluxes are all relative to the main flux at $t=0$ and the metabolite concentrations and enzyme activities are relative to their own initial values. The fraction of the main flux leaving the system via Dpl1 is taken as $\phi=0.05$ (Cungui Mao, personal communication) and the fraction of the main flux that recirculates between the free and phosphorylated sphingoid bases was arbitrarily chosen as $\psi=0.1$. The enzyme activities after heat shock were calculated from mRNA expression levels as follows. The relative mRNA induction data for heat treated yeast given in **http://genome-www.stanford.edu/clustering/Figure2.txt** was multiplied by the average of the absolute mRNA levels in untreated control cells found in two independent data sets, **http://staffa.wi.mit.edu/cgi-bin/young_public/lists.cgi?type=H&s=0** and **http://www.hsph.harvard.edu/geneexpression/.** This provided estimates of the absolute mRNA levels after heat shock (i.e. in copies per cell). These time courses were then used to form modulators of the enzyme activities as

Copyright © 2001 John Wiley & Sons, Ltd. 

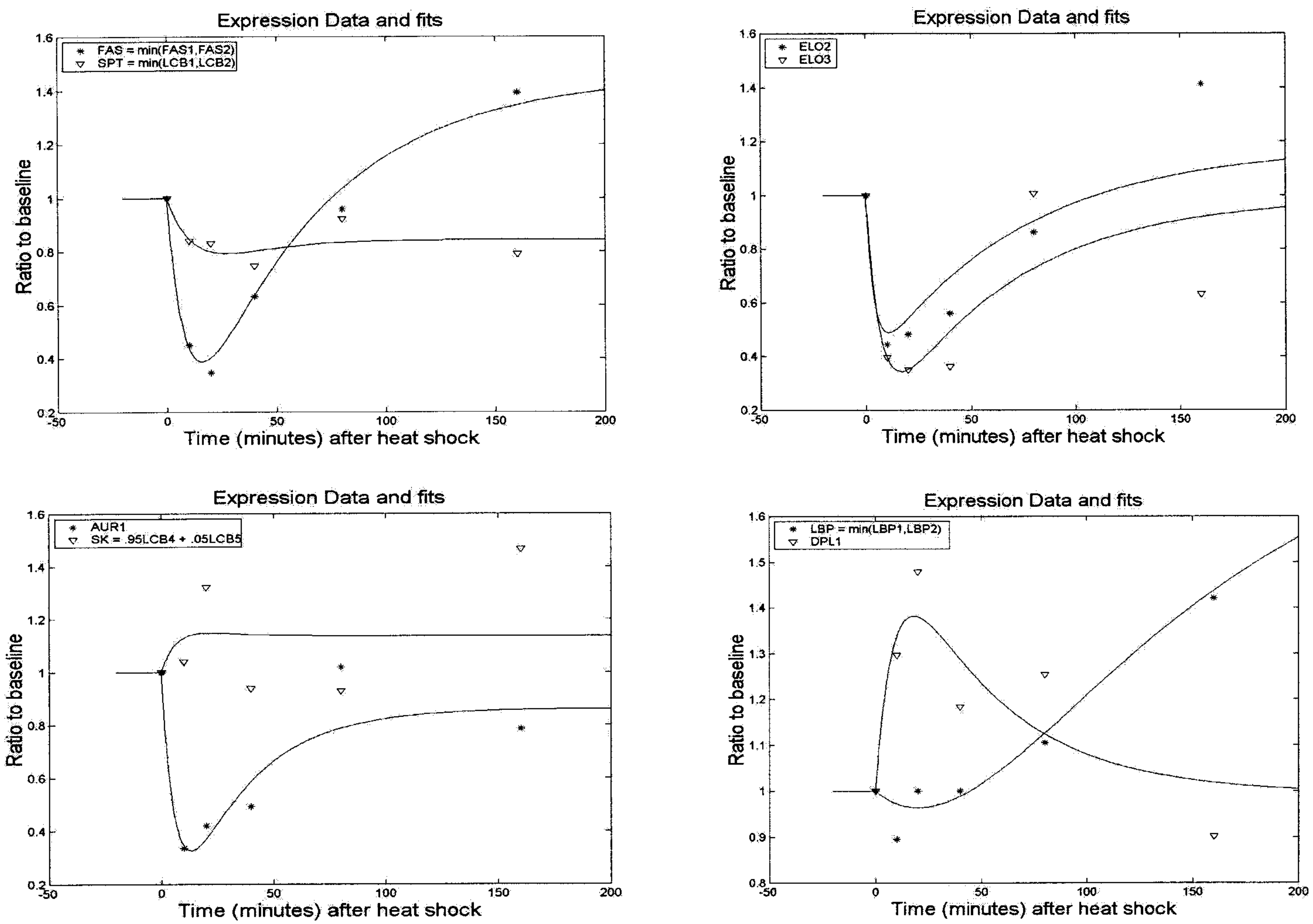

**Figure 2.** Enzyme activities from mRNA expression data. Gene expression data was modified as described in the text and each time course was fit to a 4-parameter system of two differential equations (Appendix A). Since the steady state Fas level is greater than the steady state Aur1 level, applying these fits to Equations 1–6 yields a steady state ceramide level that is above baseline, see Figure 1 and the right panels of Figures 3 and 4. Furthermore, since the steady state Elo2 level is higher than the steady state Spt level, a steady state accumulation of $C_{26}$ is expected, see Figure 1 and the left panel of Figure 3. Steady state constraints were applied to the curve fits of Fas and Dpl1 because free fits in these cases were unrealistic at large times. The gene for ceramide synthase (Cs) has not been cloned so Cs = 1 was assumed

Copyright © 2001 John Wiley & Sons, Ltd.

follows. For the heterodimer enzymes fatty acid synthase (Fas = Fas1/2), long chain base phosphatase (Lbp = Lbp1/2) and serine palmitoyl transferase (Spt = Lcb1/2), the smaller of the two mRNA expression levels evaluated at each time point was used. The results were then divided by their initial values so that each modulator is initially 1. Meanwhile, since sphingoid base kinase (Sk = Lcb4/5) is believed to exist as two independent enzymes, and since about 95% of this activity is thought to be due to Lcb4 (Nagiec *et al.*, 1998), 0.95LCB4 + 0.05LCB5 divided by its initial value was used to modulate the Sk activity. Finally, modulators of the enzyme activities for Elo2, Elo3, Aur1, and Dpl1 were formed as their mRNA levels divided by their initial values. Curve fits to the results of these data manipulations are shown in Figure 2, see Appendix A for the equations. These curve fits serve as time-varying boundary conditions that emulate the heat shock excitation environment of the biochemical system. Applying them to the model yields the metabolite time course predictions shown in Figure 3.

Immediately apparent in Figure 3 is the unbounded accumulation of $C_{26}$. To eliminate this instability without altering the predicted transients, a feedback controller (see Appendix B) was introduced to modulate the Spt activity such that the DHS level follows a target trajectory. The first part of the target trajectory is the predicted transient DHS curve of Figure 3, and the latter part is the constraint that DHS has a unit steady state. The results are shown in Figure 4 and the amount of control effort used to stabilize the system is shown in Figure 5. Figure 5 shows that the Spt activity was modulated by approximately 30% at steady state. Since this value is within the measurement error of the microarray data used, it follows that the stabilizing controller did not alter the system beyond consistency with the data.

### Discussion

The model's qualitative predictions seem to be robust; with (Figure 4) or without (Figure 3) the controller, there is a transient rise and fall of sphingoid bases followed by a permanent rise in ceramide. This prediction is qualitatively consistent with experimental results (Jenkins *et al.*, 1997; Dickson *et al.*, 1997; Skrzypek *et al.*, 1999). Quantitatively, however, the results of these experiments are not even consistent with each other; the data (not shown) are further complicated by different chain-length chemical species. The model (Equations 1–6) is based on only qualitative information (Figure 1), so its predictions are at best qualitative; if reaction rate terms $S^{0.5}$ are everywhere replaced by $S$, or $2S/(S+1)$, qualitatively, the predictions remain unchanged.

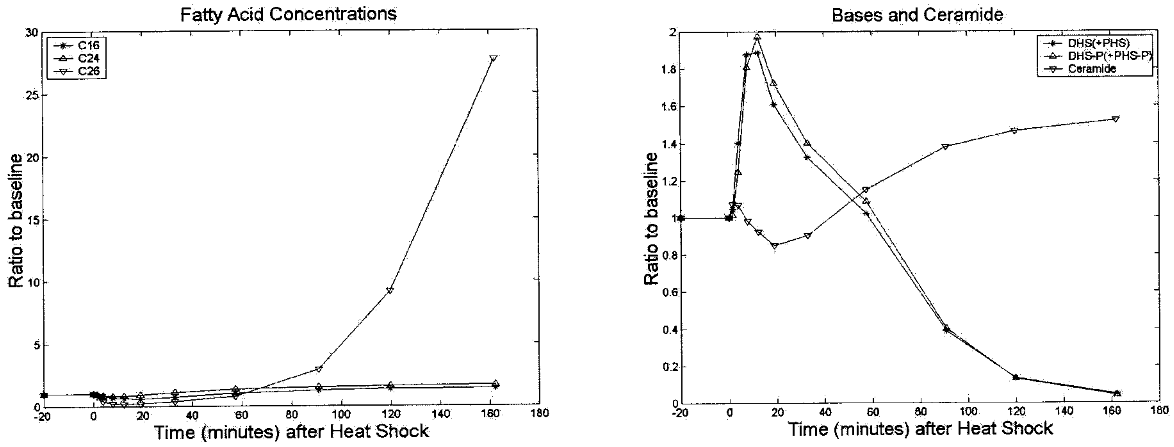

**Figure 3.** Metabolite time courses of the uncontrolled model. Unbounded growth of $C_{26}$ results because in steady state, each palmitate traversing Elo2 must be matched by at least one palmitate traversing Spt (Figure 1), but, according to the curve fits in Figure 2, the steady state flux through Elo2 is greater than the steady state flux through Spt. Here and in Figures 4 and 5, a time scale parameter $\alpha = 5$ (not shown) multiplies the right hand sides of Equations 1–6. This value is large enough that Equations 1–6 are completely stiff relative to the auxiliary equations (Appendix A) used to generate the enzyme activity time courses

Copyright © 2001 John Wiley & Sons, Ltd. 

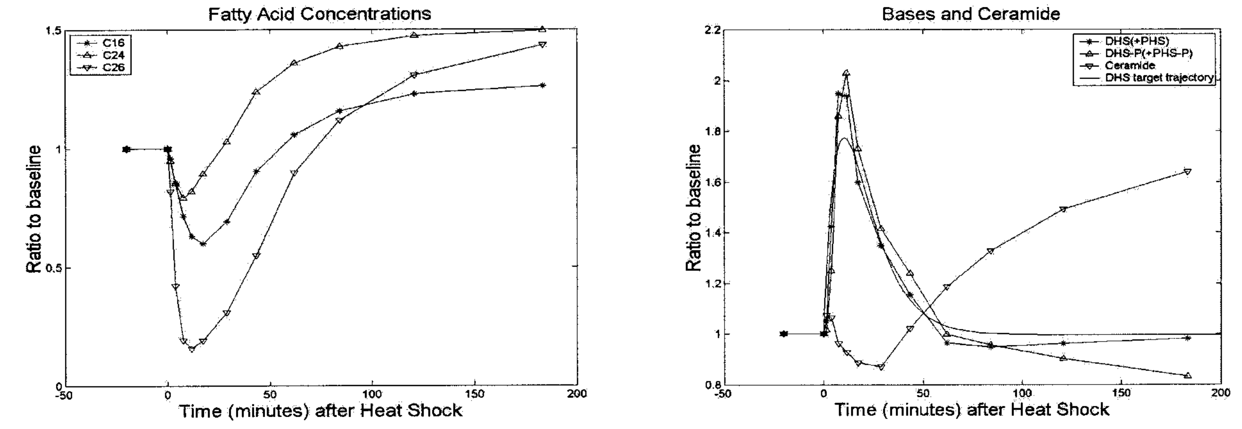

**Figure 4.** Controlled system response. The model suggests that a transient decrease in $C_{26}$ accounts for the transient rise and fall in sphingoid bases

The model also predicts, in both Figures 3 and 4, that the transient increase in sphingoid bases is perhaps the result of a transient decrease in the saturated fatty acid $C_{26}$. Since $C_{26}$ has yet to be measured in response to heat shock, and since this predicted transient decrease in $C_{26}$ was otherwise unexpected, experimental validation of this prediction would lend strong support to the hypothesis that *qualitative* metabolite time course predictions can indeed be inferred from measured mRNA levels.

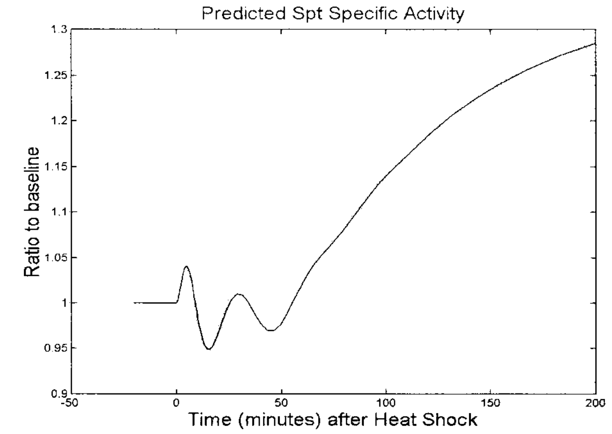

**Figure 5.** Output of the stabilizing controller. The transient behavior during the first 50 minutes is an artifact arising from an imperfect fit of the target trajectory to the predicted transient DHS response of the uncontrolled model. The controller is specified in Appendix B

### Acknowledgements

I thank the reviewers and Drs. Y. A. Hannun, R. C. Dickson, C. Mao and E. O. Voit. This publication was supported in part by funds from NIH GM57245-03.

### References

Dickson RC. 1998. Sphingolipid functions in *Saccharomyces cerevisiae*: Comparison to mammals. *Ann Rev Biochem* **67**: 27–48.

Dickson RC, Lester RL. 1999. Yeast Sphingolipids. *Biochim Biophys Acta* **1426**: 347–357.

Dickson RC, Nagiec EE, Skrzypek M, Tillman P, Wells GB, Lester RL. 1997. Sphingolipids are potential heat stress signals in *Saccharomyces*. *J Biol Chem* **272**: 30196–30200.

Eisen MB, Spellman PT, Brown PO, Botstein D. 1998. Cluster analysis and display of genome-wide expression patterns. *Proc Natl Acad Sci* **95**: 14863–14868.

Hannun YA. 1996. functions of ceramide in coordinating cellular responses to stress. *Science* **274**: 1855–1859.

Jenkins GM, Richards A, Wahl T, Mao C, Obeid L, Hannun Y. 1997. Involvement of yeast sphingolipids in the heat stress response of *Saccharomyces cerevisiae*. *J Biol Chem* **272**: 32566–32572.

Kuo BC. 1975. *Automatic Control Systems*. Prentice-Hall: Englewoods Cliffs, NJ.

Nagiec MM, Nagiec EE, Baltisberger JA, Wells GB, Lester RL, Dickson RC. 1997. Sphingolipid synthesis as a target for antifungal drugs. complementation of the inositol phosphorylceramide synthase defect in a mutant strain of *Saccharomyces cerevisiae* by the AUR1 Gene. *J Biol Chem* **272**: 9809–9817.

Nagiec MM, Skrzypek M, Nagiec EE, Lester RL, Dickson RC. 1998. The LCB4 (YOR171c) and LCB5 (YLR260w) genes of *Saccharomyces* encode sphingoid long chain base kinases. *J Biol Chem* **273**: 19437–19442.

Schneiter R. 1999. Brave little yeast, please guide us to thebes: sphingolipid function in *S. cerevisiae*. *Bioessays* **21**: 1004–1010.

Copyright © 2001 John Wiley & Sons, Ltd. 

Skrzypek MS, Nagiec MM, Lester RL, Dickson RC. 1999. Analysis of phosphorylated sphingolipid long-chain bases reveals potential roles in heat stress and growth control in *Saccharomyces*. *J Bacteriol* **181**: 1134–1140.
Voit EO. 2000. *Computational Analysis of Biochemical Systems*. Cambridge University Press: Cambridge; 158–160.

### Appendix A

The curve fits shown in Figure 2 were produced by least squares estimates of $k_1$, $k_2$, $a$ and $b$ in

$$\frac{dy}{dt} = -k_1 y(t) + ax(t)$$

$$\frac{dx}{dt} = b - k_2 x(t)$$

where $y(t)$ is fitted to the manipulated data in Figure 2 and $y(0) = x(0) = 1$. Roughly speaking, the four parameters in this model provide $y(t)$ with an amplitude, a rising time constant, a falling time constant and a steady state offset. Although analytic solutions trivially exist for this system, they are not used because the model (Equations 1–6) executes more efficiently when the time-varying boundary conditions are supplied using differential equations rather than analytic functions.

### Appendix B

The proportional-integral controller (Kuo, 1975) used to stabilize the model is given by

$$u(t) = K_p e(t) + K_i \int_0^i e(\tau) d\tau$$

where the error $e(t)$ is the difference between the target trajectory and simulated DHS levels, and where $u(t)$ modulates the Spt activity (Figure 5). With $K_p = 0$, the model (Equations 1–6) was stabilized by tuning $K_\mathrm{i}$ until DHS followed its target trajectory. This controller blatantly admits that the true (biological) mechanism of system stabilization is not known.

Copyright © 2001 John Wiley & Sons, Ltd. 
